# Fatty acid composition on diet and carcasses, growth, body indices and profile serum of Asian redtail catfish ( *Hemibagrus nemurus*) fed a diet containing different levels of EPA and DHA

**DOI:** 10.12688/f1000research.126487.2

**Published:** 2023-05-15

**Authors:** Netti Aryani, Indra Suharman, Saberina Hasibuan, Nur Asiah, Hafrijal Syandri

**Affiliations:** 1 Department of Aquaculture, Faculty of Fisheries and Marine Science, Universitas Riau, Pekanbaru, Riau, 28295, Indonesia; 2 Department of Aquaculture, Faculty of Fisheries and Marine Science, Universitas Bung Hatta, Padang, West Sumatera, 25133, Indonesia

**Keywords:** Aquaculture, Asian redtail catfish, essential fatty acids, growth rates, serum metabolites

## Abstract

**Background:** The Asian redtail catfish *Hemibagrus nemurus *is a promising commercial aquaculture freshwater big-sized Bagridae catfish across Asian countries such as the Mekong, Malay Peninsula, and Indonesia. This study analysed the effect of eicosapentaenoic acid (EPA, 20:5n-3) and docosahexaenoic acid (DHA, 22:6n-3) supplementation in diets on changes in fatty acid compositions in feed and fish meat, lipid quality (atherogenic index and thrombogenic index), growth rate, body indicators, and serum metabolites of *Hemibagrus nemurus *juveniles *. *

**Methods:** A total of 180 Asian redtail catfish (initial weight 54.80 ± 2.72 g) were fed four levels (0, 3,150, 6,300, and 9,450 mg of EPA+DHA/kg feed) sourced from fish oil. Diets were fed in triplicate in freshwater tarpaulin ponds, with 15 fish per tarpaulin pond. During the experiment, fish were fed 3% per day of the biomass weight.

**Results:** Categorically, there were significant differences in the composition of fatty acids in the feed and fish meat. The atherogenic index was between 1.76 and 1.84, and the thrombogenic index was between 0.81 and 0.89 in all fish meat. Growth performance was significantly different between diets, while body indices did not make a significant difference between diets. The fish meat EPA and DHA showed positive linear relationships with diet EPA (p <0.001, *r ^2^* = 90%) and DHA diet (p<0.001, *r ^2^* = 85%). Serum metabolites among treatments D2 and D3 diet-fed feed for 60 days did not significantly differ. Glucose (GLU) levels had moderate relationships with triglycerides (TAG) ( *r ^2^* = 65%), and GLU levels strongly correlated with low-density lipoprotein cholesterol (LDL-C) ( *r ^2^* = 81%).

**Conclusions:** Based on diets and whole-body carcass compositions, growth performance, and serum metabolites, Asian redtail catfish fed a diet containing 6,300 mg of EPA+DHA/kg feed are best for food safety.

## Introduction

Global capture fisheries production from marine sources has stagnated in the last few decades. ^1^^,^^2^ Therefore, food sources from freshwater fish farming are increasingly being recognized for their role in an environmentally sustainable and nutritionally sustainable food system. ^3^^,^^4^ However, freshwater fish contain fewer omega (ω)-3 fatty acids than omega (ω)-6 polyunsaturated fatty acids (PUFAs), making them a healthy food. ^5^^,^^6^ Therefore, fish feed freshwater must be provided with the addition of omega (ω)-3 FUPAs carried out sustainably. Several researchers have reported feed enrichment for freshwater fish, such as the addition of linolenic acid, fish oil, and soybean oil to Nile tilapia fish feed. ^6^^–^^8^ Omega (ω)-3, as well as EPA and DHA for the feed of Atlantic salmon. ^9^^,^^10^ Dietary lipid levels for juvenile Asian red-tailed catfish ( *Hemibagrus wyckioides*) and silver barb ( *Puntius gonionotus*) fingerling. ^11^^,^^12^

Aquafeed is rich in numerous significant nutrients, such as amino acids, fatty acids, vitamins, and minerals. Of these, feed is rich in nutrients and can be used by fish to increase body weight and survival, ^13^ disease resistance, and changes in the aquaculture environment. ^14^^,^^15^ This factor could also increase feed efficiency, which is usually used as a success indicator of fish farming. ^16^ However, better feed quality is related to the nutrients of the whole-body carcass and is beneficial for human health. ^1^^,^^4^^,^^5^^,^^8^^,^^14^^,^^17^

*Hemibagrus nemurus* , commonly known as “Asian redtail catfish,” is a promising commercial aquaculture freshwater big-sized Bagridae catfish across Asian countries such as Mekong, Malay Peninsula, and Indonesia. Because of its dominant consumer preference, it is most abundant in ordo Bagridae. ^18^ This species has a high growth rate, disease resistance, year-round reproduction, ^19^ and wide adaptability to the cultivation environment. ^20^^,^^21^ In recent decades, research on feed nutrition for the growth performance of Asian redtail catfish has garnered the interest of researchers, such as experiments on dietary protein levels to increase growth performance, feed efficiency, and survival rate of juveniles, ^22^^,^^23^ and the utilization of salted trash fish meal in the diet as a substitute for fish meal. ^21^ Substitution of fish meal with a mixture of by-catch and fish viscera meal mixtures. ^24^ Additionally, the addition of turmeric ( *Curcuma longa*) in artificial feed, ^25^ and addition of *Saccharomyces cerevisiae* in commercial feed, ^26^ as well as different lipid level on daily growth coefficient and feed conversion ratio of Asian redtail catfish, *Hemibagrus wyckioides*. ^11^ Until now, there has been no information on the utilities of optimum EPA and DHA levels on the sensible diet for the growth rate and fish meat value of Asian redtail catfish. We hypothesized that supplemented levels of EPA + DHA *via* fish oil in the diet could improve the nutritional quality of dietary fatty acid and fish meat, feed efficiency, growth rate, body indices, and lipid profiles in the serum of Asian redtail catfish. Therefore, the first aim of this study was to investigate the effect of the EPA and DHA levels in diets on the fatty acid composition. The second aim was to analyse fish meat fatty acids, growth rate, feed efficiency, and nutritional quality of lipids.

## Methods

### Ethical statement

This study was conducted under the project entitled ‘Optimization of the use of EPA- and DHA-type fatty acids in the feed of Asian redtail catfish to strengthen food security after coronavirus disease (COVID-19)’. This research was funded by the Ministry of Education, Culture, Research, and Technology of the Republic of Indonesia, which has been approved by the Institute for Research and Community Service, Universitas Riau (grant letter: 1633/UN19.5.1.3/PT.01.03/2022). The Ethics Community Research and Community Service Universitas Riau approved collecting and rearing of *Hemibagrus nemurus* juveniles with the ARRIVE guidelines, which have been stated in the letter of grant No. 1633/UN19.5.1.3/PT.01.03/2022, May 11, 2022. Additionally, according to Indonesian Legislation, the Asian redtail catfish were not categorized as a protected species. The research was carried out in the Hatchery Laboratory, Faculty Fisheries and Marine, Riau University for 60 days from May to June 2022. This study is reported in line with the ARRIVE guidelines. ^27^ We have made efforts to alleviate the suffering of experimental animals in this study, except for some euthanized animal experiments that were carried out by piercing part of the fish brain. The total number of fish in the study was 180 fish consisting of 45 fish for each treatment. Each treatment consisted of 15 fish per replicate (replicate = 3). A total of three fish were anesthetized in each replicate for the analysis of whole-body flesh, and body indices. Before the fish was euthanized, it was soaked in fresh water at a temperature of 10°C for five minutes. The goal is for the fish to be calmer and pierce their brain easier. After that, the fish were put to sleep on a board with a cork bottom, and their brain was pierced with a large animal syringe (9G × 1 inch). Then the fish were dissected to measure their body indices. In addition, the flesh was collected to measure its fatty acid composition.

### Preparation experiment diet

Asian redtail catfish adapted for 30 days to standard, 2 mm pellet feed. The dietary composition (% dry weight) consisted of an 8.76% moisture level, 34.52% crude protein, 5.94% crude fat, 41.13% carbohydrates, 9.65% ash content and 3.85% crude fibre ( Table 1). The total calories were 356.06 Kcal/100 g vitamin D (328.31 mcg/100 g). Minerals consisted of calcium (1,788.11 mg/100 g), phosphorus (1,081.764 mg/100 g), magnesium (298.42 mg/100 g), manganese (9.50 mg/100 g), sodium (549.30 mg/100 g), and zinc (14.10 mg/100 g).

**Table 1.** List of ingredients and analysis of experimental diets containing 0, 0.5, 1 and 1.5% fish oil.

Ingredients (%)	D1	D2	D3	D4
Fish meal	25	25	25	25
Soybean meal	27	27	27	27
Rice bran	41	40.5	40	39.5
Fish oil	0	0.5	1	1.5
Corn oil	3	3	3	3
CMC (carboxy methyl cellulose)	2	2	2	2
Vitamin-Mineral Mix	1	1	1	1
Cellulose	1	1	1	1
	100	100	100	100
Proximate composition (dry weight)				
Moisture	8.64	9.04	8.67	9.17
Ash	9.58	9.42	9.61	9.59
Crude Protein	34.54	34.53	33.72	33.75
Crude Fat	5.90	6.42	7.57	7.69
Crude fibre	3.82	3.96	3.88	3.69
NFE	46.22	45.57	45.37	45.22
Energy (Kcal/g)	356.54	358.18	364.71	363.01

Note: nitrogen-free extract (NFE) = 100 - (% crude protein + % crude fat + % ash + % crude fibre).

The vitamin mix contains vitamins A, 2,750 IU; Vit D, 550,000 IU; Vit E, 25,000 IU; Ca D-pantothenate, 25,000 mg, Mg, 25.000 mg; P, 50.000 mg.

After adaptation, the fish were fed supplemented with fish oil (EPA + DHA) at a dosage of 0 g/kg feed (labelled D1). A total of 5 g/kg feed, consisting of 1,950 mg EPA and 1,200 mg DHA (labelled D2). The 10 g/kg feed consisted of 3,900 mg EPA and 2,400 mg DHA (labelled D3). The 15 g/kg feed consisted of 5,850 mg EPA and 3,600 mg DHA (labelled D4). EPA and DHA were mixed manually to the aquafeed to produce quality feed, which was then given to the animal experiment.

### Experimental procedure and sampling

Fish samples were weighed using the AD-600i, with an accuracy of 0.01 g (ACIS model number AD-600i, China). The Indonesian Directorate of Metrology has approved the use of ACIS model AD-600i. Additionally, body length was measured with a measuring board with an accuracy of 1 mm. A total of 180 juvenile *Hemibagrus nemurus* were counted (initial body weight was 54.80 ± 2.72 g, and initial body length was 15.77 ± 2.5 cm). The age of the fish was 120-day post-hatching (120 DPH), whose sex has not been determined. Juveniles were obtained from the Hatchery Laboratory Faculty of Fisheries and Marine Science Universitas Riau. This species' health status was good, not a hybridization genetic modification. A total of 12 round tarpaulin ponds (diameter = 80 cm, height = 60 cm, water depth is 50 cm with a water volume of 400 litres) were placed in Hatchery Laboratory and equipped with continuous aeration. This experiment consisted of four treatments and three replicates, and each round tarpaulin pond was stocked with 15 juveniles randomly stocked. The Asian redtail catfish were fed pellets 2 mm in size supplemented with EPA and DHA, namely, D1, D2, D3, and D4 diets. Daily feeding was performed at 07:00, 13:00, 18:00, and 22:00 at a body weight rate of 3% per day for 60 experimental days. A feeding rate of 3% has been optimal for the growth of Hemibagrus nemurus in this study, although some researchers reported a range of 3 - 8%. Every 20 days, five fish samples were taken from each tarpaulin pond. Before sampling, the fish fasted for 12 hours to vacate their visceral contents. Fish samples were anesthetized orally using clove oil, and body weight was measured to determine the amount of fed feed. Then, their weights were measured. After that, the fish were returned to their respective tarpaulin ponds according to treatment and replication.

### Fatty acid analysis

The feed and whole-body meat of the fish of each treatment were examined utilizing a fatty acid composition by gas chromatography-mass spectrometry (GC-MS). The extraction of the total lipid was carried out according to the method described by Folch *et al.* (1957), as explained by Rajion ^28^ utilizing a chloroform: methanol (2.1. v/v) solvent system. Transmethylation was carried out using 14% methanolic boron trifluoride. Transmethylation was carried out using 14% methanolic boron trifluoride. The derivatized fatty acid methyl esters (FAMEs) were separated on a Quadrex 007 series bonded phase fused silica capillary column (Quadrex Corporation, New Haven. CT, USA) (30 m × 0.25 mm ID, 0.20 mm film thickness, 007 Carwax/BTR) in a 5890 Hewlett-Packard Gas-Liquid Chromatograph (Hewlett-Packard Co., Avondale, PA). Individual fatty acids were recognized and measured by proportion with retention time and peak areas of FAMEs standards (Supelco 37 Component FAME mix and Nu-Check Prep Inc., GLC-569). The fatty acid composition of feed and whole-body meat of Asian redtail catfish were examined by Saraswanti Indo Genetech Laboratory, Bogor-Indonesia (SIG Laboratory, Accredited Testing Laboratory- LP -184-IDN).

### Tarpaulin pond water quality

The water quality values of the round tarpaulin ponds that were used to rear the Asian redtail catfish juveniles were recorded weekly. Water quality was measured at 10:00 AM at a distance inward of 10 cm from the water surface of each tarpaulin pond to detect the water temperature, dissolved oxygen, and pH. A thermometer (Celsius scale) was used to measure water temperature. Water oxygen (O2; mg/L) was measured by an oxygen metre (YSI Model 52, Yellow Instrument Co, Yellow Spring, OH, USA). The pH value of the water was calculated on a digital pH metre (Mini 0–14 pH IQ, Scientific Chemo Science Thailand). Additionally, we also measured the level of nitrate-nitrogen (NO3-N; mg/L), total alkalinity (mg/L), and hardness (mg/L) calculated according to standard American Public Health Association (APHA) procedures. ^29^

### Calculations

All fish from each treatment and replicate (n=15 fish per replicate) were measured in length and weighed body weight separately for the final experiment. Final length (FL), final weight (FW), weight gain (WG, %), and specific growth rate (SGR, %/day). The feed conversion ratio (FCR) and survival rate (SR) were analysed using the support formula as follows:

$$\text{Weight gain}\;\left(\%\right)=\frac{\text{Final body weight}\;\left(g\right)-\text{Initial body weight}\;\left(g\right)}{\text{Initial body weight}\;\left(g\right)}\times 100$$

$$\;\text{Specific growth rate}\;\left(\%/\mathrm{day}\right)=\frac{\ln\;\text{Final body weight}\;\left(g\right)-\ln\;\text{Initial body weight}\;\left(g\right)}{\text{Culture}\;\mathrm{day}}\times 100$$

$$\text{Feed conversion ratio}=\frac{\text{Feed supply in}\;\mathrm{kg}}{\text{Total harvest weight in}\;\mathrm{kg}}$$

$$\text{Survival rate}=\frac{\text{The final number of fish}}{\text{The initial number of fish}}\times 100$$

For the analysis of the body indices of Asian redtail catfish ( *Hemibagrus nemurus*), three fish were sacrificed each in the tarpaulin pond, and their weight and length were measured. After that, it was immediately dissected to determine the Condition Factor (CF), Hepatosomatic Index (HSI), Viscerosomatic Index (VSI), and Liposomatic Index (LSI) as given below:

$$\text{Condition Factor}=\frac{\text{Body weight of the fish}\;\left(g\right)}{\text{The body length of the fish}\;{\left(\mathrm{cm}\right)}^{3}}\times 100$$

$$\text{Hepatosomatic Index}=\frac{\text{Liver weight of the fish}\;\left(g\right)}{\text{Body weight of the fish}\;\left(g\right)}\times 100$$

$$\text{Viscerosomatic Index}=\frac{\text{Viscera weight of the fish}\;\left(g\right)}{\text{Body weight of the fish}\;\left(g\right)}\times 100$$

$$\text{Liposomatic Index}=\frac{\text{Visceral}\;\mathrm{fat}\;\text{weight of the fish}\;\left(g\right)}{\text{Body weight of the fish}\;\left(g\right)}\times 100$$

The nutritional quality of lipid AI and TI was calculated based on the equations ^30^

$$\text{Atherogenic Index}\;\left(\mathrm{AI}\right)=\frac{\left[\left(C12:0+4+C14:0+C16:00\right)\right]}{\left[\left(\sum \text{MUFA}+\sum n-6+\sum n-3\right)\right]}$$

$$\text{Thrombogenic Index}\;\left(\mathrm{TI}\right)=\frac{\left[\left(C,14,:,0,+,C,16,:,0,+,C,18,:,0\right)\right]}{\left[\left(0.5\times \sum \text{MUFA}\right)+\left(0.5\times \sum n-6\right)+\left(3\times \sum n-3\right)+\left(\sum n-3/\sum n-6\right)\right]}$$

Where:

C12:0 = Lauric acid

C14:0 = Meristic acid

C16:0 = Palmitic acid

C18:0 = Stearic acid

∑MUFA = Sum concentrations of all unsaturated fatty acid

∑n-6 = Sum concentrations of n-6 polyunsaturated fatty acid

∑n-3 = Sum concentrations of n-3 polyunsaturated fatty acid

### Lipid profiles in serum

In the present study, to minimize stress on the experimental animals, the animals within the containers were lightly anesthetized with 1 ml/10 L clove oil for 2–3 min until the loss of coordination was visible. Afterward, the blood was collected by puncturing the caudal vertebrae tail vessels using a hypodermic needle of 1 ml (made in Indonesia). Briefly, blood samples from each specimen were placed in an Eppendorf tube, and then centrifuged at 3,000 rpm for 15 minutes (5804R, Eppendorf), and the supernatant serum rather than plasma was stored at -21°C. Then it was analysed at the Centre for Primate Animal Studies at IPB University, Bogor, Indonesia. Serum glucose (GLU) was analysed using the glucose oxidase method using commercial kit (Pathology Laboratory, the Centre for Primate Animal Studies, IPB University, Bogor, Indonesia), Primate Animal Study Center, IPB University-Bogor, has standardized commercial kits to measure blood fish parameters. Triglycerides (TAG) were measured using the GPO-PAP method, total cholesterol (TC) by the CHOP-PAP method, high-density lipoprotein (HDL) with direct method-select inhibition method, low-density lipoprotein (LDL) with direct method-surfactant removal were also measured using commercial investigation kit (Pathology Laboratory, the Centre for Primate Animal Studies, IPB University, Bogor, Indonesia), and the ratio of LDL-C and HDL-C were approximated as outlined previously. ^31^

### Data analysis

To determine the trial effect of supplemented EPA and DHA in diets, body meat, growth performance, body indices, and serum metabolite variables were measured using one-way ANOVA. The data from the experiment were analysed using SPSS (RRID:SCR_002865) 16.0 software package (SPSS, Chicago, IL). The data homogeneity was analysed with Levin's test and followed up with the post hoc Duncan’s multiple range test. ^32^ Relationship between dietary EPA and DHA with whole-body meat; glucose levels, TAG levels, and LDL-C levels were analysed using Regression with curve estimation. For the figures presented, Microsoft Office Professional Plus 2019 was used.

## Results

### Fatty acid concentrations in the experimental diet

In this study, the ∑MUFA in the four diets showed higher values than ∑SAFA and ∑PUFA. Regarding the ∑MUFA, the D1 diet had the highest value, followed by the D2, D3, and D4 diets with C-oleic acid (C18:1, n-9) abundance in all diets. The SAFA palmitic acid (C16:0) and stearic acid (C18:0) were abundant in all diets. However, in the PUFA, the higher composition was linoleic acid (C18:2 n-6) ( Table 2). ^27^

**Table 2.** Fatty acid profile (% of total FA) and total lipids in the diet supplemented with the EPA and DHA.

Fatty acids	Fish oil	Experiment diets				P-Value	SE ^1^
		D1	D2	D3	D4		
C12:0, Lauric	nd	0.220 ^a^	0.233 ^b^	0.200 ^c^	0.220 ^ad^	0.619	0.024
C14:0, Myristic	1.31	1.590 ^a^	1.593 ^b^	1.633 ^c^	1.590 ^d^	0.063	0.015
C15:0, Pentadecanoic	0.17	0.173 ^a^	0.177 ^b^	0.170 ^c^	0.167 ^d^	0.400	0.005
C16:0, Palmitic	10.84	25.363 ^a^	27.540 ^b^	27.550 ^c^	26.713 ^d^	0.000	0.197
C17:0, Margaric	1.23	0.350 ^a^	0.388 ^b^	0.417 ^c^	0.427 ^d^	0.000	0.007
C18:0, Stearic	3.95	4.973 ^a^	4.993 ^b^	4.903 ^c^	3.490 ^d^	0.000	0.023
C20:0, Arachidic	0.40	0.350 ^a^	0.033 ^b^	0.337 ^c^	0.257 ^d^	0.035	0.027
C21:0, Heneicocylic	0.10	0.053 ^a^	0.050 ^b^	0.057 ^c^	0.070 ^d^	0.132	0.007
C23:0, Tricosylic	nd	0.050 ^a^	0.033 ^b^	0.020 ^c^	0.021 ^d^	0.001	0.004
C24:0, Lignoceric	nd	0.117 ^a^	0.087 ^b^	0.107 ^c^	0.103 ^d^	0.023	0.007
C14:1, Myristoleic	0.15	0.043 ^a^	0.070 ^b^	0.110 ^c^	0.150 ^d^	0.000	0.007
C16:1, Palmitoleic	3.62	1.913 ^a^	1.900 ^b^	1.950 ^c^	1.420 ^d^	0.074	0.192
C17:1, cis10 Heptadecanoic	0.79	0.177 ^a^	0.187 ^b^	0.200 ^c^	0.223 ^d^	0.000	0.005
C18:1, Oleic	15.47	34.130 ^a^	33.477 ^b^	32.850 ^c^	32.167 ^d^	0.000	0.066
C20:1, Paullinic	5.29	0.610 ^a^	0.680 ^b^	0.760 ^c^	0.833 ^d^	0.000	0.024
C20:2, Dihomo-linoleic	0.23	0.283 ^a^	0.263 ^b^	0.250 ^c^	0.233 ^d^	0.002	0.008
C22:1 n-9, Erucic	7.74	0.053 ^a^	0.583 ^b^	1.023 ^c^	0.140 ^d^	0.000	0.008
C22:2, cis-13,16 Docosadienoic	0.11	0.300 ^a^	0.183 ^b^	0.180 ^c^	0.140 ^d^	0.000	0.007
C18:2 n-6, Linoleic	2.74	19.487 ^a^	19.843 ^b^	18.770 ^c^	18.033 ^d^	0.000	0.102
C18:3 n-6, Gamma-Linolenic	0.28	0.140 ^a^	0.113 ^b^	0.147 ^c^	0.137 ^d^	0.007	0.007
C20:3 n-6, Dihomo-gamma-linolenic	0.14	0.147 ^a^	0.147 ^b^	0.137 ^c^	0.137 ^d^	0.370	0.007
C20:4 n-6, Arachidonic	1.53	0.463 ^a^	0.530 ^b^	0.547 ^c^	0.550 ^d^	0.000	0.007
C18:3 n-3, α-Linolenic	1.48	1.890 ^a^	1.970 ^b^	1.923 ^c^	1.863 ^d^	0.000	0.012
C20:3 n-3, Eicosatrienoic	0.10	0.067 ^a^	0.067 ^b^	0.060 ^c^	0.050 ^d^	0.109	0.006
C20:5 n -3, EPA	23.82	1.320 ^a^	2.023 ^b^	2.850 ^c^	3.387 ^d^	0.000	0.014
C22:6 n-3, DHA	18.28	2.270 ^a^	2.503 ^b^	3.048 ^c^	3.123 ^d^	0.000	0.029
Σ n-3	43.89	6.147 ^a^	6.563 ^b^	7.882 ^c^	8.823 ^d^	0.000	0.050
Σ n-6	4.69	20.237 ^a^	20.633 ^b^	19.600 ^c^	18.857 ^d^	0.000	0.111
Σn-6/Σn-3	0.10	3.292 ^a^	3.144 ^b^	2.487 ^c^	2.137 ^d^	0.000	0.019
DHA/EPA	1.30	2.174 ^a^	1.237 ^b^	1.070 ^c^	1.040 ^d^	0.000	0.009
EPA+DHA	42.1	4.190 ^a^	4.527 ^b^	5.898 ^c^	6.910 ^d^	0.000	0.041
∑SFA	18.08	33.090 ^a^	35.440 ^b^	35.483 ^c^	33.140 ^d^	0.109	1.143
∑MUFA	33.57	38.777 ^a^	37.360 ^b^	37.315 ^c^	36.817 ^d^	0.000	0.245
∑PUFA	48.68	26.38383 ^a^	27.197 ^b^	27.489 ^c^	27.680 ^d^	0.000	0.139
∑FA	100	100 ^a^	100 ^b^	100 ^c^	100 ^d^	0.000	0.038
Lipid content (%)		5.937 ^a^	6.49 ^b^	7.64 ^c^	7.75 ^d^	0.265	1.448

Notes: 0 g fish oil/kg feed (labelled D1); 5 g fish oil/kg feed, consisted of 1,950 mg EPA and 1,200 mg DHA (labelled D2); 10 g fish oil/kg feed consisted of 3,900 mg EPA and 2,400 mg DHA (labelled D3); and 15 g fish oil/kg feed consisted of 5,850 mg EPA and 3,600 mg DHA (labelled D4).

Means ± SE (standard error) of three separate determinations, a b c d = significant in a row, nd = not detected

FA, fatty acid; DHA, docosahexaenoic acid; EPA, eicosapentaenoic acid; SFA, saturated fatty acids; MUFA, monounsaturated fatty acids; PUFA, polyunsaturated fatty acids.

### Fatty acid concentrations in body meat

**Figure 1.**
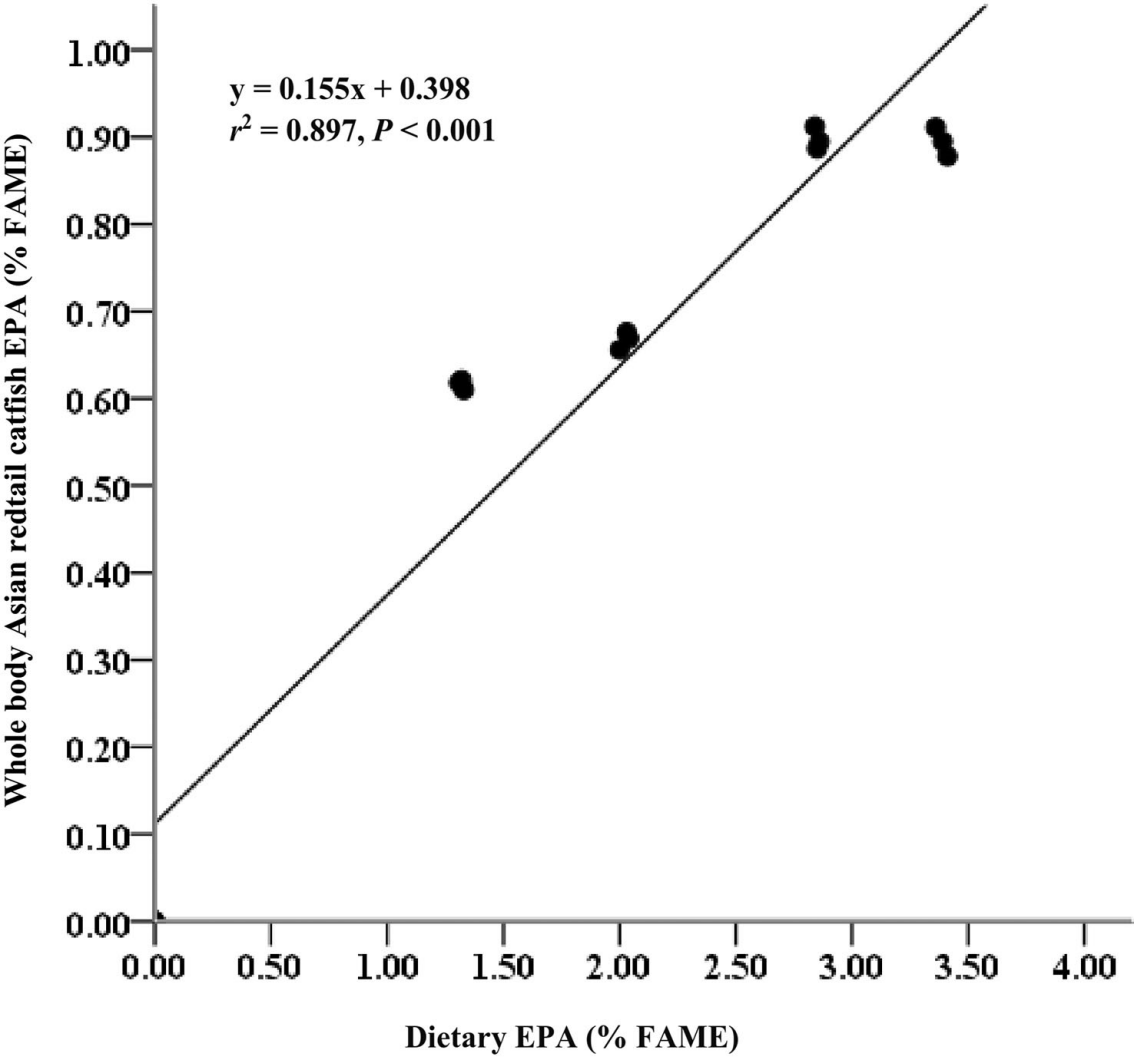
Relationship between dietary EPA content (% FAME) and whole body EPA content of Asian redtail catfish, feed diets containing increasing EPA levels over 60 days. EPA, eicosapentaenoic acid; FAME, fatty acid methyl ester.

**Figure 2.**
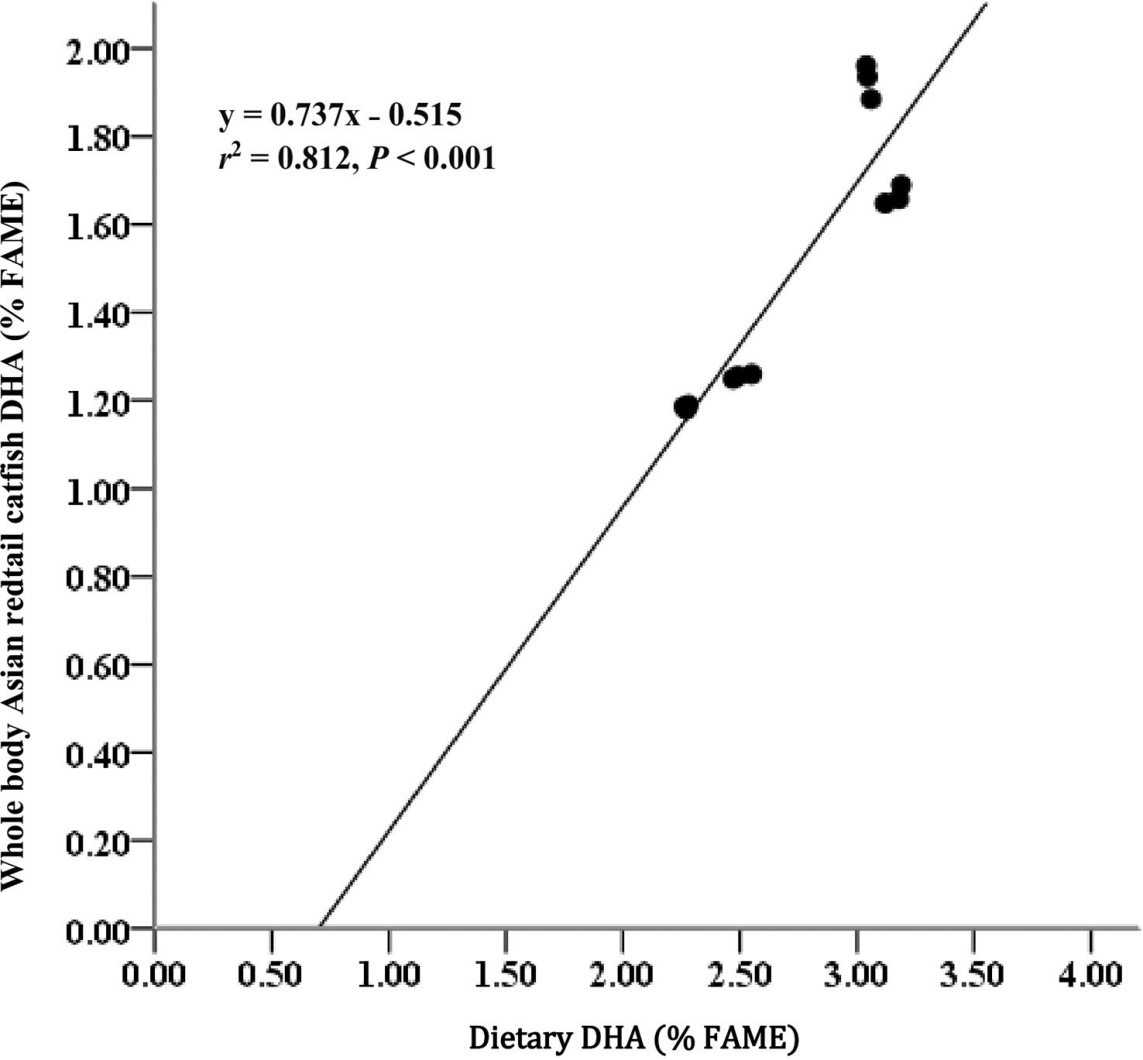
Relationship between dietary DHA content (% FAME) and whole body DHA content of Asian redtail catfish feed diet containing increasing DHA levels over 60 days. DHA, docosahexaenoic acid; FAME, fatty acid methyl ester.

**Figure 3.**
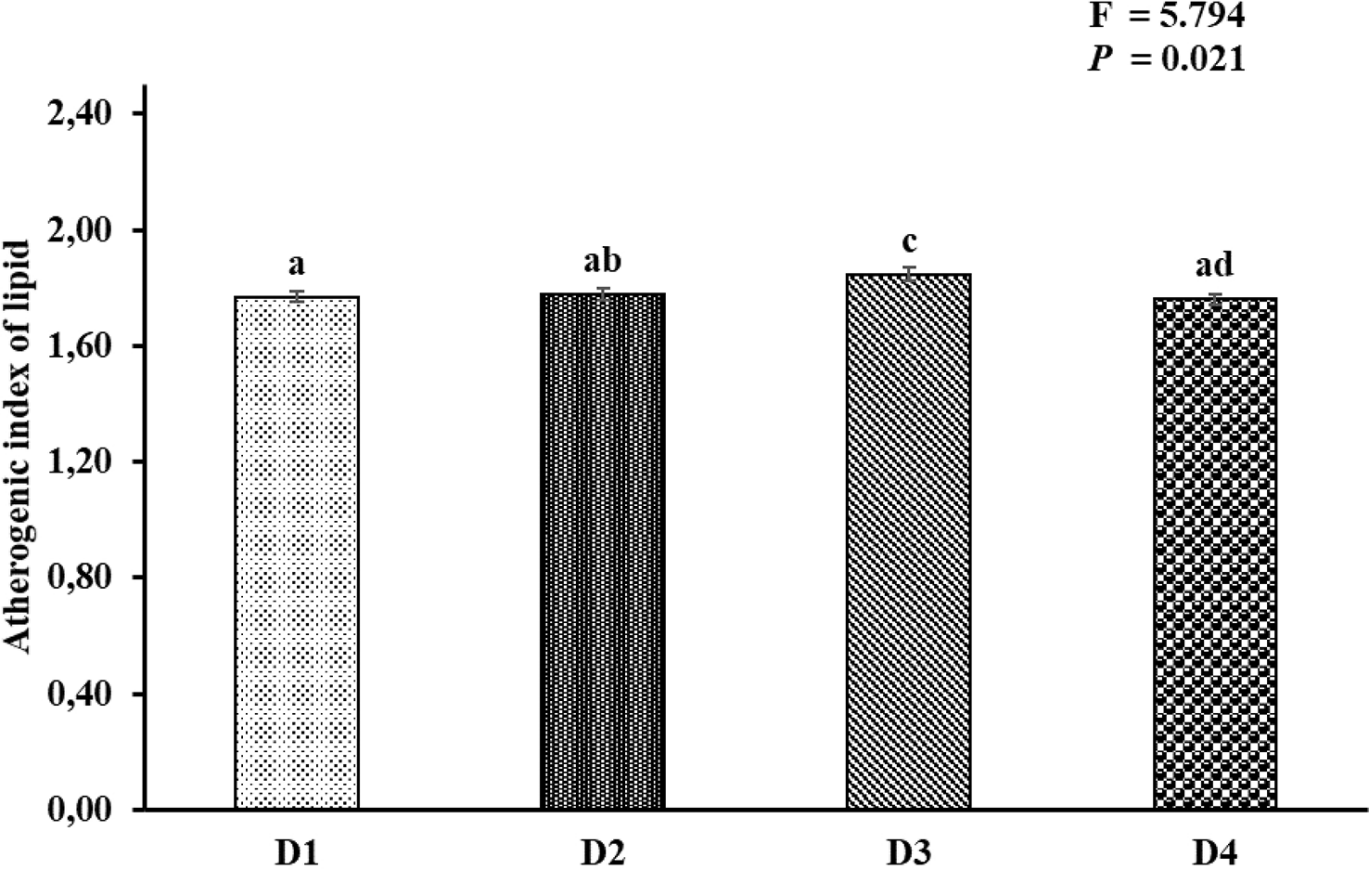
Nutritional quality of lipid atherogenic index of Asian redtail catfish whole body containing EPA and DHA levels over 60 days. EPA, eicosapentaenoic acid; DHA, docosahexaenoic acid. Note: A different superscript letter on each bar chart indicates a significant difference (P < 0.05). The same superscript letter on each bar chart does not show a significant difference (P > 0.05).

**Figure 4.**
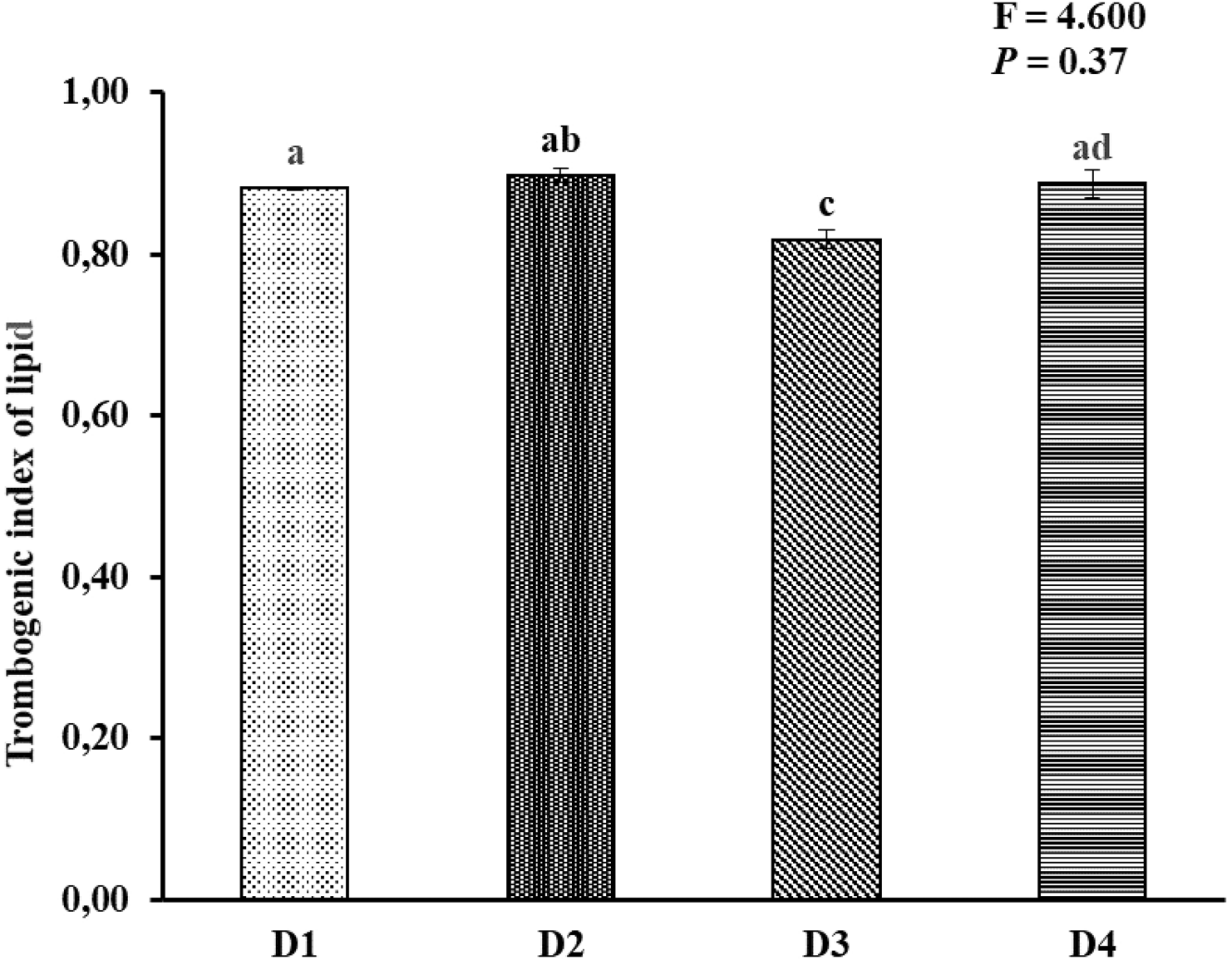
Nutritional quality of lipid thrombogenic index of Asian redtail catfish whole body containing EPA and DHA levels over 60 days. EPA, eicosapentaenoic acid; DHA, docosahexaenoic acid. Note: A different superscript letter on each bar chart indicates a significant difference (P < 0.05). The same superscript letter on each bar chart does not show a significant difference (P > 0.05).

The Asian redtail catfish fed feed D1, D2, D3, and D4 showed higher levels of ∑MUFA in the body meat than in ∑SAFA and ∑PUFA. Regarding MUFA, oleic acid (C18:1) is the most abundant. Palmitic acid (C16:0) and stearic acid (C18) were significant concentrations of SAFAs. Conversely, linoleic acid (C18:2, n-6) abounds in PUFA. We noted that EPA and AA concentrations were lacking in all body meat, while DHA was higher ( Table 3). EPA and DHA levels in feed have strong relationships with EPA and DHA levels in body meat ( *r* ^2^ = 0.897 for EPA; Figure 1 and *r* ^2^ = 0.812 for DHA; Figure 2). AI and TI were significantly higher on the D3 diet than on the other treatments. Duncan's post hoc test showed that the AI and TI for fish fed D1 were not significantly different ( *p* > 0.05) from those provided for D2 and D4 ( Figures 3 and 4).

**Table 3.** Fatty acid concentrations (% of total FA) of the Asian redtail catfish ( *Hemibagrus nemurus*) fed with different diets after the 60 days reared.

Fatty acids	Experiment diets				P-value	SE ^1^
	D1	D2	D3	D4		
C12:0, Lauric	0.760 ^a^	0.614 ^b^	0.773 ^c^	0.730 ^d^	0.000	.007
C14:0, Myristic	2.277 ^a^	1.881 ^b^	2.367 ^c^	2.372 ^cd^	0.000	.027
C15:0, Pentadecanoic	0.169 ^a^	0.155 ^b^	0.181 ^c^	0.181 ^cd^	0.002	.004
C16:0, Palmitic	27.266 ^a^	25.480 ^b^	25.824 ^c^	26.783 ^d^	0.000	.093
C17:0, Margaric	0.248 ^a^	0.245 ^ab^	0.285 ^c^	0.245 ^ad^	0.000	.026
C18:0, Stearic	8.156 ^a^	8.566 ^b^	9.226 ^c^	9.271 ^cd^	0.000	.133
C20:0, Arachidic	0.244 ^a^	0.252 ^b^	0.306 ^c^	0.253 ^bd^	0.000	.029
C24:0, Lignoseric	0.109 ^a^	0.078 ^b^	0.112b ^c^	0.053 ^d^	0.000	.001
C16:1, Palmitoleic	2.177 ^a^	1.938 ^b^	2.348 ^c^	2.413 ^cd^	0.000	.036
C17:1, cis 10 Heptadecanoic	0.162 ^a^	0.154 ^b^	0.182 ^c^	0.183 ^d^	0.000	.002
C18:1, Oleic	37.565 ^a^	35.097 ^b^	35.342 ^c^	36.439 ^d^	0.000	.303
C20:1, Paullinic	0.839 ^a^	0.750 ^b^	1.025 ^c^	0.935 ^d^	0.000	.006
C20:2, Dihomo-linoleic	0.677 ^a^	0.614 ^b^	0.748 ^c^	0.656d	0.000	.009
C22:2,cis 13,16 Docosadienoic	1.093 ^a^	1.500 ^b^	nd	nd	0.000	.011
C18:2 n-6, Linoleic	12.874 ^a^	11.668 ^b^	14.603 ^c^	13.726 ^d^	0.000	.172
C18:3 n-6, Gamma-Linolenic	1.271 ^a^	0.345 ^b^	0.369 ^c^	0.404 ^d^	0.000	.004
C20:3 n-6, Dihomo-Gamma-linolenic	0.913 ^a^	0.816 ^b^	1.011 ^c^	0.930 ^d^	0.000	.012
C20:4 n-6, Arachidonic	0.465 ^a^	0.425 ^b^	0.503 ^c^	0.496 ^d^	0.000	.005
C18:3 n-3, α-linolenic	0.853 ^a^	0.768 ^b^	1.006 ^c^	0.895 ^d^	0.000	.126
C20:3 n-3, Eicosatrienoic	nd	0.813 ^b^	0.907 ^c^	nd	0.000	.011
C20:5 n -3, EPA	0.616 ^a^	0.667 ^b^	0.898 ^c^	0.895 ^cd^	0.000	.009
C22:6 n-3, DHA	1.186 ^a^	1.255 ^b^	1.927 ^c^	1.665 ^d^	0.000	.020
Σ n-3	2.655 ^a^	3.503 ^b^	4.738 ^c^	3.455 ^d^	0.000	.038
Σ n-6	15.524 ^a^	15.255 ^b^	15.487 ^c^	15.557 ^d^	0.420	.188
Σn-3: Σn-6	0.171 ^a^	0.230 ^b^	0.306 ^c^	0.222 ^d^	0.000	.029
DHA/EPA	1.924 ^a^	1.881 ^b^	2.146 ^c^	1.862 ^d^	0.000	.030
EPA+DHA	1.802 ^a^	1.922 ^b^	2.825 ^c^	2.560 ^d^	0.000	.023
∑SAFA	39.231 ^a^	37.272 ^b^	39.074 ^ac^	39.890 ^d^	0.000	.166
∑MUFA	42.514 ^a^	40.053 ^b^	39.645 ^bc^	40.626 ^d^	0.000	.325
∑PUFA	18.179 ^a^	16.758 ^b^	21.225 ^c^	19.012 ^d^	0.000	.222
∑FA	99.925 ^a^	94.083 ^b^	99.943 ^c^	99.527 ^d^	0.000	.040
Lipid content	9.200 ^a^	9.380 ^b^	11.567 ^c^	10.400 ^d^	0.000	.461

Notes: 0 g fish oil/kg feed (labelled D1); 5 g fish oil /kg feed, consisted of 1,950 mg EPA and 1,200 mg DHA (labelled D2); 10 g fish oil/kg feed consisted of 3,900 mg EPA and 2,400 mg DHA (labelled D3); and 15 g fish oil/kg feed consisted of 5,850 mg EPA and 3,600 mg DHA (labelled D4).

FA, fatty acid; DHA, docosahexaenoic acid; EPA, eicosapentaenoic acid; SFA, saturated fatty acids; MUFA, monounsaturated fatty acids; PUFA, polyunsaturated fatty acids.

^1^ Means ± SE (standard error) of three separate determinations, a b c d = significant in a row, nd= not detected

### Growth performance and body indices

All experimental diets given to Asian redtail catfish presented a significant (p < 0.05) effect on final body weight, body weight gain (%), and specific growth rate (%/day). The higher growth was contributed by the D3 diet, followed by a better feed conversion ratio (FCR). The mean values of CF, HSI, VIS, and LSI did not differ significantly (p > 0.05) among the D1, D2, D3, and D4 diets ( Table 4). The survival rate of various diet experiments was insignificant (p > 0.05).

**Table 4.** Growth performance and body indices of Asian redtail catfish ( *Hemibagrus nemurus*) during the 60-day experimental period.

Parameters	D1	D2	D3	D4	P-value	SE ^1^
FL (cm)	19.43	19.64	19.97	20.03	0.411	0.463
FW (g)	92.412 ^a^	103.242 ^b^	110.097 ^c^	101.499 ^d^	0.002	2.770
WG (%)	63.983 ^a^	81.269 ^b^	91.952 ^c^	75.663 ^d^	0.003	4.860
SGR (%/day)	0.824 ^a^	0.991 ^b^	1.086 ^c^	0.937 ^c^	0.002	0.039
FCR	1.988 ^a^	1.633 ^b^	1.469b ^c^	1.737 ^d^	0.002	0.087
SR (%)	85.925	86.666	88.888	86.666	0.902	4.190
CF (%)	0.988	0.981	1.030	0.922	0.839	0.119
HSI (%)	0.950	0.942	1.150	1.110	0.690	0.214
VSI (%)	0.901	1.071	0.969	0.947	0.816	0.181
LSI (%)	0.191	0.510	0.240	0.190	0.175	0.148

^1^ Values are presented as mean ± SE, n = 3. Note: Numbers followed by different superscript of letters in the same row indicate a significant differences (P < 0.05). Numbers followed by superscript of the same letter in the same row showed no significant difference (P > 0.05). FL, final length; FW, final body weight; WG, weight gain; SGR, specific growth rate; FCR, feed conversion ratio; SR, survival rate; CF, condition factor; HSI, hepatosomatic index; VSI, viscerosomatic index; LSI, liposomatic index.

### Lipid profile in serum

**Figure 5.**
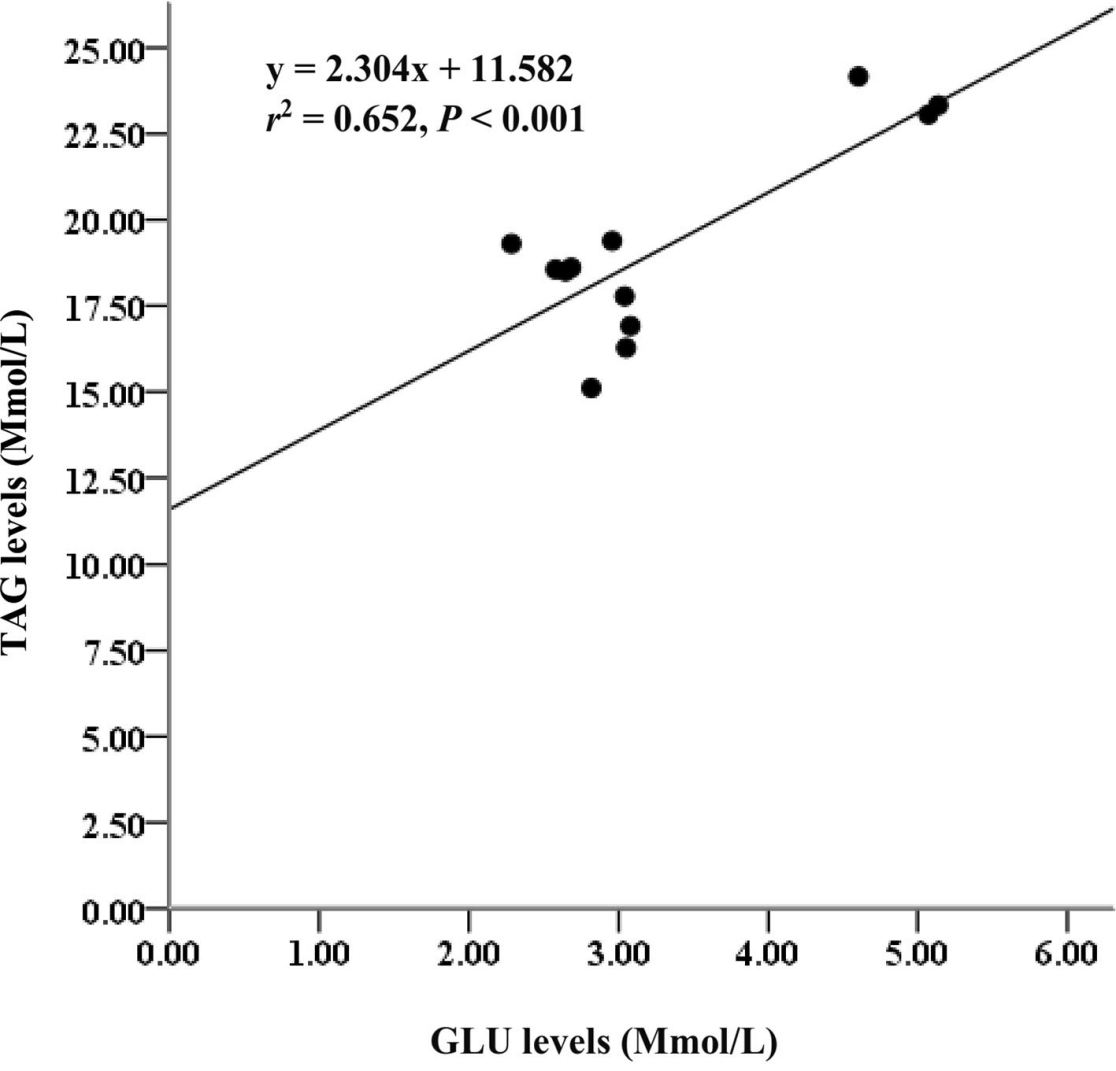
Relationship between GLU and TAG levels and Asian redtail catfish fed diet containing EPA + DHA over 60 days. GLU, glucose; TAG, triglycerides; EPA, eicosapentaenoic acid; DHA, docosahexaenoic acid.

**Figure 6.**
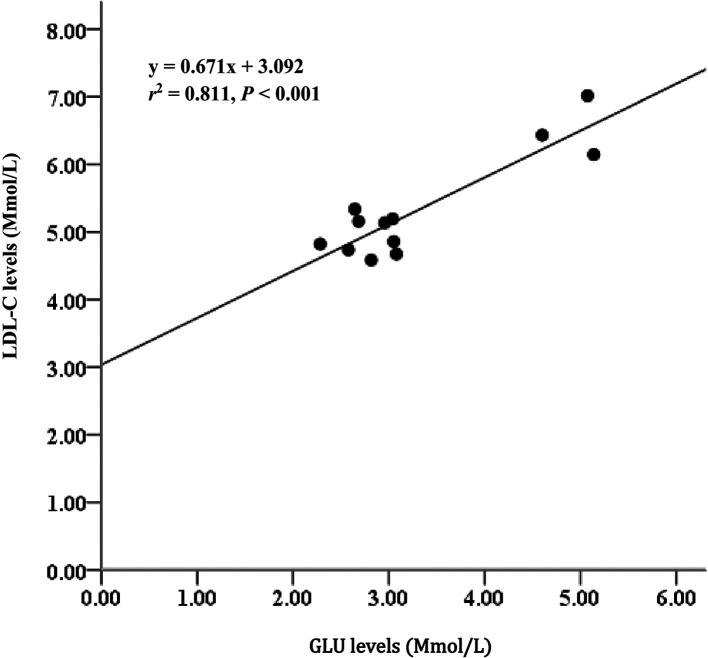
Relationship between GLU and LDL-C levels and Asian redtail catfish fed diet containing EPA + DHA over 60 days. GLU, glucose; LDL-C, low-density lipoprotein cholesterol; EPA, eicosapentaenoic acid; DHA, docosahexaenoic acid.

The serum GLU, TAG, TC, HDL-C, and LDL-C levels in fish fed a diet with no supplemental fish oil (control, D1) were significantly higher than those in fish-fed diets D2, D3, and D4. Glucose levels in experimental animals fed D2 and D3 and D3 and D4 showed no significant difference (p > 0.05), except for D2 and D3 diets. Additionally, LDL-C and HDL-C ratios indicated a significant effect (p <0.05) among the experimental diets ( Table 5). The GLU level parameter also had moderate relationships with TAG ( *r* ^2^ = 0.652, Figure 5). Additionally, the GLU level parameter strongly correlated with LDL-C ( *r* ^2^ = 0.811, Figure 6).

**Table 5.** Serum metabolites (Mmol/L) of Asian redtail catfish ( *Hemibagrus nemurus*) juveniles fed an experimental diet for 60 days.

Serum metabolites	D1	D2	D3	D4	P-value	SE ^1^
GLU	4.94 ^a^	2.50 ^b^	2.89 ^bc^	2.98 ^cd^	0.000	3.103
TAG	23.52 ^a^	18.79 ^b^	18.59 ^bc^	16.10 ^d^	0.000	10.454
TC	9.92 ^a^	8.50 ^b^	8.44 ^bc^	7.00 ^d^	0.000	5.675
HDL-C	4.03 ^a^	3.20 ^b^	3.35 ^bc^	2.85 ^d^	0.001	3.129
LDL-C	6.53 ^a^	4.97 ^b^	5.60 ^bc^	4.71 ^d^	0.001	5.003
LDL-C/HDL- C ratio	1.62 ^a^	1.55 ^b^	1.67 ^c^	1.65 ^d^	0.000	0.002

^1^ Values are presented as means ± SE, n = 3. Note: Numbers followed by different superscript of letters in the same row indicate a significant differences (P < 0.05). Numbers followed by superscript of the same letter in the same row showed no significant difference (P > 0.05). GLU, glucose; TAG, triglycerides; TC, total cholesterol; HDL-C, high-density lipoprotein-cholesterol; LDL-C, low-density lipoprotein cholesterol.

### Pond water quality

The physicochemical water parameters in the tarpaulin ponds for rearing juvenile Asian redtail catfish were as follows: water temperatures ranged from 28 to 30°C, oxygen between 6.4 and 6.7 mg/L, and pH between 6.6 and 6.8. The water alkalinity increased from 60.5 to 67.5 mg/L HCO _3_, and the hardness varied between 67.5 and 69.5 mg/L HCO _3_.

## Discussion

For food safety and to increase the efficiency of feed use in fish farming, it is necessary to use nutrient-rich feed, such as fatty acids, amino acids, minerals, and vitamins. ^7^^,^^33^^,^^34^ According to previous research, commercial feeds do not contain complete nutrients such as EPA and DHA. ^4^ Therefore, improving the nutrition of fish feed can be done by supplementing fish oil containing EFA and DHA. In this study, adding fish oil to feed directly correlated with the EPA and DHA composition for the D1, D2, D3, and D4 diets. The higher the level of addition of fish oil (EFA and DHA) to the feed, the higher the levels of EPA and DHA in the experimental diets.

Additionally, in all experimental diets, the MUFA level was higher than the SAFA and PUFA levels. By contrast, Nile tilapia feed supplemented with fish oil at 5, 10, and 15% showed higher SAFA levels than MUFA and PUFA levels. ^7^ However, EPA and DHA supplementation in Atlantic salmon feed were 0.25, 0.50, 0.75, and 2%, and SAFA levels were lower than MUFA and PUFA levels. By contrast, EPA + DHA in feed was detected in 1.35, 3.33, 5.48, and 15.6% of the total fatty acid methyl ester (FAME). ^10^ In this study, EPA and DHA addition to D1, D2, D3, and D4 diets were 0 g fish oil/kg feed, 5 g fish oil (consisted of 1,950 mg EPA and 1,200 mg DHA/kg feed), 10 g fish oil (consisted of 3,900 mg EPA and 2,400 mg DHA/kg feed), and 15 g fish oil (consisted of 5,850 mg EPA and 3,600 mg DHA/kg feed). The EFA + DHA values detected in the experimental feed D1, D2, D3, and D4 were 4.19, 4.53, 5.90, and 6.91%, respectively. Seemingly, EPA and DHA were detected in the D1 diet, even though it’s not added with fish oil, but levels were lower than D2, D3, and D4. However, it has been reported that EPA and DHA were not detected in commercial pellet feed. ^4^ Conversely, EFA and DHA were found in commercial fish feed, which enriches coconut water and palm sap sugar, fermented with various fungi.

The ratios of DHA and EPA are determined by the levels of DHA and EPA in the feed. In this study, DHA/EPA ratios between 1.05 and 2.17 were lower in the D4 and D3 diets than in D1 and D2. In this context, a ratio of 1.05 in the D4 diet indicated that DHA and EPA were at their highest levels in the experimental diet. In this study, the DHA/EPA ratio differences within the experimental diet were due to the addition of EPA and DHA at different levels. Zhang *et al.* ^35^ state that the diet's proportion of DHA/EPA must be precise to develop better feed formulations. For this case, the DHA/EPA ratio of the diet for maximum growth of Nile tilapia was 1.42, ^7^ including 1.70 for giant gourami ^4^ and 0.53 for Atlantic salmon. ^10^ Supplementation with fish oil in aquafeed directly reflects the chemical composition of aquafeed and can influence DHA/EPA ratios. ^10^^,^^36^ Arachidonic acid (ARA), EPA, and DHA are the main essential HUFAs in fatty acid compositions, and these elements may be preventively supplemented in the diet. ^37^ In general, certain types of fatty acids are principal energy sources and crucial constructional portions for the diet. ^38^

In this study, the fish feed supplemented with 10 g of fish oil (consisting of 3,900 mg EPA and 2,400 mg DHA/kg of feed) had the highest fat content in the whole-body carcass, 11.567%, with 39.074% SAFAs, 39.645% MUFAs, and 21.225% PUFAs. By contrast, the fat level in whole body carcasses fed the D1 diet with the lowest fat content was 9.20% with 39.231% SAFA, 42.514% MUFA, and 18.179% PUFA, respectively. This study found that MUFA levels were higher than SAFA levels in the all-body carcass of Asian redtail catfish fed fish D1, D2, D3, and D4. In Nile tilapia-fed pellets with fish oil added at 0, 5, 10, and 15% for 60 days, MUFA levels were also higher than SAFAs and PUFAs. ^7^ However, Atlantic salmon in freshwater-fed feed were enriched at levels of 0.25, 0.5, 0.75, and 2.0% of EFA + DHA, and their whole-body carcasses also contained higher MUFA levels than SAFAs and PUFAs. ^10^ By contrast, in wild specimens, sea bass ( *Dicentrarchus labrax*) showed the highest level of PUFA compared with SAFAs and MUFA. ^39^ In common carp ( *Cyprinus carpio*), Rohu ( *Labeo rohita*), and tilapia ( *Oreochromis niloticus*) specimens captured in the Indus River, Pakistan, the levels of SAFA were higher than those of MUFA and PUFA. ^40^ The total SAFA, MUFA, and PUFA in body meat depends on the supplemented fish oil, levels of DHA+EPA in the diet, and fish species. Additionally, it also depends on whether the body meat is from farmed fish or wild catch. Whether the SAFA, MUFA, and PUFA levels of Asian redtail catfish from cultured and wild captured sources are different is poorly understood.

Palmitic acid (C16:0) was the major metabolite in the body meat, followed by stearic acid (C18:0). Palmitic and stearic acid levels were higher in fed fish D4. Oleic acid (C18:1) was identified as the essential MUFA in the fish carcass fed D1, D2, D3, and D4. The higher oleic acid (C18:1) in all carcasses could be due to its dominance in the pellet feed. Several authors reported that commercial fish feeds contain more oleic acid. ^4^^,^^7^^,^^39^

The current study showed that Asian redtail catfish ( *Hemibagrus nemurus*) fed feed supplemented with different levels of EPA and DHA obtained from fish oil contained higher EPA and DHA than control feed (D1). These results match those for other fish species. ^4^^,^^6^^,^^7^ The EPA and DHA contents in the diet had strong relationships with the EPA and DHA contents in whole-body carcasses ( *r* ^2^ = 0.897 for EPA and *r* ^2^ = 0.812 for DHA). This fact showed that adding EPA and DHA to the feed positively contributed to the EPA and DHA in the carcass of Asian redtail catfish. Regarding PUFAs, Asian redtail catfish freshwater can be considered a good source of n-3 series fatty acids, especially EPA and DHA, which have the highest content in fish fed a D3 diet. However, the levels were low, in line with the contents of EPA and DHA for Atlantic salmon freshwater fish, ^10^ Nile tilapia, ^7^ and common carp, Rohu, and tilapia. ^40^ However, this level of EPA and DHA in freshwater finfish is lower than that in seawater finfish, such as sea bass and other fish species. ^5^^,^^39^

EPA and DHA are essential nutrients in a portion of healthy food, so finding an affordable source of PUFA from fish is crucial for consumer guidance because fish is one of the significant sources of EPA and DHA. The ratio of Σn-3: Σn-6 fatty acids was higher in fish fed D3 than in fish fed D1, D2, and D4, which showed that EPA and DHA supplements in the diet can improve carcass quality. Fish oil is one of the low-cost supplies of EPA and DHA. It is essential to aquafeed, especially in freshwater fish farming, such as Asian redtail catfish ( *Hemibagrus nemurus*).

In the current study, the atherogenic index (AI) ranged from 1.76 to 1.84, and the thrombogenic index ranged from 0.81 to 0.89 in all whole-body carcasses fed feed D1, D2, D3, and D4. This appeared to be connected to a discrepancy in SAFA compositions among experimental diets. The AI levels in the whole-body carcasses of Asian redtail catfish fed feed D1 and D2 and D4 were insignificant, while fish fed feed D3 differed in D1, D2, and D4. The AI and thrombogenic index (TI) indices were significantly associated with the content of myristic acid (C14:0), palmitic acid (C16:0), and stearic acid (C18:0), all of which are thrombogenic promotors. ^7^ The Food Agricultural Organization and the Health World Organization recommend AI and TI values between 0.4 and 0.5. Even though the AI and TI scores of the Asian redtail catfish were higher than 0.5, we stated that consuming Asian redtail catfish flesh is a healthy food. In fish farming, AI and TI indices correlated with feed supplemented with fish oil, including EPA and DHA, ^7^^,^^10^ feed quality used, ^4^ fish species, and environmental factors. ^5^^,^^41^

Fish feeds with different levels of EPA and DHA directly impact the growth rate of Asian redtail catfish juveniles. In this study, the final body weight, body weight gain (%), specific growth rate (%/days), and low feed conversion ratio were shown in fed fish D3. In this trial, enrichment pellet feed with EPA and DHA has a positive effect on the fatty acid composition of the feed. This factor causes the better growth rate of Asian redtail catfish. The SAA, MUFA, and PUFA contents were higher in feed supplemented with EPA and DHA. Higher fat levels in the D3 diet also increased the growth rate of *Hemibagrus nemurus* and have been observed in other species. ^4^^,^^10^

In this study, feed supplemented with EPA and DHA at different levels sourced from fish oil did not affect the condition factor (CF) or body indices (HSI, VSI, LSI) of Asian redtail catfish during the 60-day experimental period. The results were the same as those for *Micropterus salmoides* and other species. ^42^^–^^45^

The nutritional status and metabolism of reared fish indirectly reflect the blood biochemistry of fish. The protein, carbohydrate, and fat contents in food can change the levels of GLU, TAG, HDL, and LDL in blood serum and are closely linked to the activity of digestive enzymes. In this study, we did not analyze digestive enzyme activity, so the discussion related to the levels of GLU, TAG, HDL, and LDL in blood serum cannot get discussed yet; maybe in the future can be developed. A consistent GLU level is one of the crucial indicators of human health. ^34^ In the current study, the amount of GLU in the blood serum of fish was lower in fish fed the D2, D3, and D4 diets than in fish fed the D1 diet, with a constant level of GLU between 2.50 and 2.98 Mmol/L. However, TAG is the usual variety of fat that can be utilized behind time by the body for energy. ^46^ The present study showed a difference in the serum TC, HDL-C, LDL-C levels, and LDL-C/HDL-C ratios, except between the D2 and D3 diets. These results indicated that EPA and DHA levels up to 9,400 mg/kg feed were conducive to energy storage for fish health and can be recommended for consumer food safety. This finding can minimize feed costs overall, whether it is fish oil or future sources of EPA + DHA, because the aquaculture sector needed 836 thousand tonnes of fish oil. ^47^

## Conclusions

Asian redtail catfish ( *Hemibagrus nemurus*) diet containing 6,300 mg of EPA + DHA *via* fish oil (diet D3) showed fatty acid compositions in the diets better than those other feed diets. The D3-fed feed to *Hemibagrus nemurus* also had a better effect on the fatty acid composition of body meat, nutritional quality of lipid AI and TI, growth rate, body indices, and serum metabolites. AI and TI values in the meat of Asian redtail catfish at 6,300 mg of EPA+DHA were 0.81 and 1.85. Foods with low AI and TI promote excellent human health, for example, by preventing human health coronary diseases. According to our research, the current inclusion of EPA and DHA *via* fish oil in fish feed is approximately 9,450 mg EPA + DHA/kg diet, which could be reduced as much as 3,150 mg EPA + DHA/kg diet. This finding could minimize the overall cost of aquafeed, whether it is fish oil or future sources of EPA + DHA.

## Data Availability

Figshare: Fatty acid composition on diet and whole body, growth performance, body indices, and profile blood serum of Asian redtail catfish ( *Hemibagrus nemurus*) fed a diet containing different levels of EPA and DHA. https://doi.org/10.6084/m9.figshare.21164425. ^27^ This project contains the following underlying data:
-
Table 1. Raw data list ingrediennts and proximate of feed.pdf -
Table 2. Raw data fatty acid of experiment diet.pdf -
Table 3. Raw data fatty acid of whole body of Asian redtail catfish over 60 daysdocx.pdf -
Table 4. Raw data initial body weight of Asian redtail catfish.pdf -
Table 5. Raw data initial body length of Asian redtail catfish.pdf -
Table 6. Raw data final body weight of Asian redtail catfish each experimental diet.pdf -
Table 7. Raw data final body length of Asian redtail catfish each experimental diet.pdf -
Table 8. Raw Data of growth performance and body indices of Asian redtail catfish.pdf -
Table 9. Raw data serum metabolites of Asian redtail catfishdocx.pdf - Authors ceklist Manuscriot No. 126487.pdf (completed ARRIVE checklist) Data are available under the terms of the Creative Commons Attribution 4.0 International license (CC-BY 4.0).

## References

[1] Tran N Rodriguez Chan CY : Indonesian aquaculture futures: An analysis of fish supply and demand in Indonesia to 2030 and role of aquaculture using the AsiaFish model. *Mar. Policy.* 2017;79:25–32. 10.1016/j.marpol.2017.02.002

[2] FAO: *The state of food security and nutrition in the world.* Rome: Food and Agriculture Organization of the United Nations;2018.

[3] Syandri H Azrita A Sumiarsih E : Nutrient loading and farm characteristics of giant gourami fish aquaculture systems in Lake Maninjau, Indonesia: basic knowledge of production performance [version 2; peer review: 2 approved]. *F1000Res.* 2021;10:378. 10.12688/f1000research.52613.2 34621506 PMC8459621

[4] Undefined A , Syandri H Aryani N : The utilization of new products formulated from water coconut, palm sap sugar, and fungus to increase nutritional feed quality, feed efficiency, growth, and carcass of gurami sago (Osphronemus goramy Lacepède, 1801) juvenile [version 1; peer review: 1 approved with reservations]. *F1000Res.* 2021;10:1121. 10.12688/f1000research.74092.1

[5] Mohanty BP Mahanty A Ganguly : Nutritional composition of food fishes and their importance and providing food and nutritional security. *Food Chem.* 2019;293:561–570. 10.1016/j.foodchem.2017.11.039 31151648

[6] Chen C Guan W Xie Q : n-3 essential fatty acids in Nile tilapia, Oreochromis niloticus: Bioconverting LNA to DHA is relatively efficient, and the LC-PUFA biosynthetic pathway is substrate limited in juvenile fish. *Aquaculture.* 2018;495:513–522. 10.1016/j.aquaculture.2018.06.023

[7] Duarte FOS Faula FG de Prado GS : Better fatty acid profile in fillets of Nile tilapia *(Oreochromis niloticus)* supplemented fish oil. *Aquaculture.* 2020;534:736241. 10.1016/j.aquaculture.2020.736241

[8] Godoy AC Santos OO Oxford JH : Soybean oil for Nile tilapia *(Oreochromis niloticus)* in finishing diets: Economic, zootechnical and nutritional meat improvements. *Aquaculture.* 2019;512:734324. 10.1016/j.aquaculture.2019.734324

[9] Huyben D Grobler T Matthew C : Requirement for omega-3 long-chain polyunsaturated fatty acids by Atlantic salmon is relative to the dietary lipid level. *Aquaculture.* 2021;531:735805. 10.1016/j.aquaculture.2020.735805

[10] Qian C Hart B Colombo SM : Re-evaluating the dietary requirement of EPA and DHA for Atlantic salmon in freshwater. *Aquaculture.* 2020;518:734870. 10.1016/j.aquaculture.2019.734870

[11] Deng J Zhang X Sun Y : Optimal dietary lipid requirement for juvenile Asian red-tailed catfish *(Hemibagrus wyckioides)*. *Aquac. Rep.* 2021;20:100666. 10.1016/j.aqrep.2021.100666

[12] Nayak M Saha A Pradhan A : Influence of dietary lipid levels on growth, nutrient utilization, tissue fatty acid composition and desaturase gene expression in silver barb *(Puntius gonionotous)* fingerlings. *Comp. Biochem. Physiol. B: Biochem. Mol. Biol.* 2018;226:18–25. 10.1016/j.cbpb.2018.08.005 30118764

[13] Syandri H Azrita : Enrichment of commercial feed with new formula products on the growth, yield, and mortality of the giant gourami *Osphronemus goramy*. *IOP Conf. Ser.: Earth Environ Sci.* 2022;1062:012007. 10.1088/1755-1315/1062/1/012007

[14] Deng Y Mao C Lin Z : Nutrients, temperature, and oxygen mediate microbial antibiotic resistance in sea bass *(Lateolabrax maculatus)* pond. *Sci. Total Environ.* 2022;819:153120. 10.1016/j.scitotenv.2022.153120 35041966

[15] Xia R Hao Q Xie Y : Effects of dietary Saccharomyces cerevisiae on growth, intestinal and liver health, intestinal microbiota and disease resistance of channel catfish *(Ictalurus punctatus)*. *Aqua Rep.* 2022;24:101157. 10.1016/j.aqrep.2022.101157 35569779

[16] Henriksson PJG Troell M Banks LK : Interventions for improving the productivity and environmental performance of global aquaculture for future food security. *One Earth.* 2021;4:1220–1232. 10.1016/j.oneear.2021.08.009

[17] Lall SP Dumas A : 3-Nutritional requirements of cultured fish: formulating nutritionally adequate feeds. *Feed and Feeding Practices in Aquaculture (Second Edition).* 2022;66–132. 10.1016/B978-0-12-821598-2.00005-9

[18] Aryani N Suharman I Azrita A : Diversity and distribution of fish fauna of upstream and downstream areas at Koto Panjang Reservoir, Riau Province, Indonesia [version 2; peer review: 2 approved]. *F1000Res.* 2020;8:1435. 10.12688/f1000research.19679.2 32117566 PMC7029753

[19] Aryani N Suharman I : Effects of 17ß-estradiol on the Reproduction of Green Catfish *(Hemibagrus nemurus, Bagridae)*. *Fish. Aquac. J.* 2014; 5;1:163–166.

[20] Aryani N Suharman I : effect of dietary protein level on the reproductive performance of female of green catfish *(Hemibagrus nemurus Bagridae)*. *J Aquac Res Development.* 2015;6:377. 10.4172/2155-9546.1000377

[21] Hasan B Iskandar P Indra S : Growth performance and carcass quality of river catfish Hemibagrus nemurus fed salted trash fish meal. *Egypt. J. Aquat. Res.* 2019;45(3):259–264. 10.1016/j.ejar.2019.07.005

[22] Suhenda N Samsudin R Nugroho E : Growth of green catfish *(Hemibagrus nemurus)* fry in floating net cage feed by artificial food with different protein content. *Jurnal Iktiologi Indonesia.* 2010;10(1):67–71.

[23] Deng J Zhang X Bi B : Dietary protein requirement of juvenile Asian red-tailed catfish Hemibagrus wyckioides. *Anim. Feed Sci. Technol.* 2011;170(3-4):231–238. 10.1016/j.anifeedsci.2011.08.014

[24] Kusmini II Kurniawan K Putri FP : Analysis of growth and nutritional values of three generations of Asian redtail catfish *(Hemibagrus nemurus)*. *AACL Bioflux.* 2020; 13;6:3348–3359.

[25] Sukendar W Pratama WW Anggraini SI : Growth Performance of Baung (Hemibagrus nemurus) Provided with Artificial Feed with Addition of Turmeric *(Curcuma longa Linn.)*. *AquaMarine.* 2021;8(1):8–13.

[26] Rachmawati D Elfitasari T Samidjan I : Protein digestive performance, feed utilization efficiency and growth of Sangkuriang Catfish *(Clarias Gariepinus Var Sangkuriang)* through supplementation of Saccharomyces Cerevisiae in commercial feed. *J. Indonesia. Aquac.* 2021;5(2):216–222. 10.14710/sat.v5i2.11684

[27] Aryani N Suharman I Hasibuan S : Fatty acids composition on diet and whole body, growth performance, body indices and profile blood serum of Asian redtail catfish (Hemibagrus nemurus) fed a diet containing different levels of EPA and DHA. figshare.[Dataset].2022. 10.6084/m9.figshare.21164425 PMC10233315 37273964

[28] Rajion MA : Essential fatty acid metabolism in the fetal and neonatal lamb. PhD. Thesis. The University of Melbourne Australia. 1985.

[29] APHA: *Standard Methods for Examination of Water and Wastewater.* Washington DC, USA: American Public Health Association; 19th ed 1995.

[30] Ulbricht CHT Southgate DAT : Coronary heart disease: Seven dietary factors. *Lancet (London).* 1991;338(8773):985–992. 10.1016/0140-6736(91)91846-M 1681350

[31] Friedewald WT Levy RI Fredrickson DS : Estimation of the concentration of low-density lipoprotein cholesterol in plasma, without use of the preparative ultracenrifuge. *Clin. Chem.* 1972;18:499–502. 10.1093/clinchem/18.6.499 4337382

[32] Duncan DB : Multiple ranges and multiple F tests. *Biometrics.* 1955;11:1–42. 10.2307/3001478

[33] Hua K Cobcroft JM Cole A : The future of aquatic protein: Implications for protein sources in aquaculture diets. *One Earth.* 2019;1(3):316–329. 10.1016/j.oneear.2019.10.018

[34] Zhang Q Zhang Y Zhang X : Effects of dietary florfenicol contained feeds on growth and immunity of European seabass *(Dicentrarchus labrax)* in flow-through and recirculating aquaculture system. *Aqua Rep.* 2021;19:100602. 10.1016/j.aqrep.2021.100602

[35] Zhang M Chen C You C : Effect of different dietary ratios of docosahexaenoic to eicosapentaenoic acid (DHA/EPA) on the growth, non-specific immune indices, tissue fatty acid compositions, and expression of genes related to LC-PUFA biosynthesis in juvenile golden pompano *(Trachinotus ovatus)*. *Aquaculture.* 2019;505:488–495. 10.1016/j.aquaculture.2019.01.061

[36] Dupont-Cyr BA Le François NR Christen F : Linseed oil as a substitute for fish oil in the diet of Arctic charr *(Salvelinus alpinus)*, brook charr *(S. fontinalis)* and their reciprocal hybrids. *Aqua Rep.* 2022;22:100949. 10.1016/j.aqrep.2021.100949

[37] Turchini GM Francis DS Keast RSJ : Transforming salmonid aquaculture from a consumer to a producer of long-chain omega-3 fatty acids. *Food Chem.* 2011;124:609–614. 10.1016/j.foodchem.2010.06.083

[38] Rodrigues de Souza ML Gasparino E Reis Goes ES dos : Fish carcass flours from different species and their incorporation in tapioca cookies. *Future Food: J. Food Agric. Soc.* 2022;5:100132. 10.1016/j.fufo.2022.100132

[39] Fuentes A Fernández-Segovia I Serra JA : Comparison of wild and cultured sea bass *(Dicentrarchus labrax)* quality. *Food Chem.* 2010;119(4):1514–1518. 10.1016/j.foodchem.2009.09.036

[40] Jabeen F Chaudhry SA : Chemical compositions and fatty acid profiles of three freshwater fish species. *Food Chem.* 2011;125(3):991–996. 10.1016/j.foodchem.2010.09.103

[41] Luzia LA Sampaio GR Castellucci CMM : The influence of season on the lipid profiles of five commercially important species of Brazilian fish. *Food Chem.* 2003;83(1):93–97. 10.1016/S0308-8146(03)00054-2

[42] Yadav AK Rossi W Habte-Tsion HM : Impacts of dietary eicosapentaenoic acid (EPA) and docosahexaenoic acid (DHA) level and ratio on the growth, fatty acid composition, and hepatic-antioxidant status of largemouth bass *(Micropterus salmoides)*. *Aquaculture.* 2020;529:735683. 10.1016/j.aquaculture.2020.735683

[43] Arriaga-Hernàndez D Crisantema H Martínez-Montaño E : Fish meal replacement by soybean products in aquaculture feeds for white snook, Centropomus viridis: Effect on growth, diet digestibility, and digestive capacity. *Aquaculture.* 2021;530:735823. 10.1016/j.aquaculture.2020.735823

[44] Hassan HU Ali QM Ahmad N : Assessment of growth characteristics, the survival rate and body composition of Asian sea bass Lates calcarifer (Bloch, 1790) under different feeding rates in a closed aquaculture system. *Saudi J. Biol. Sci.* 2021; 28;28:1324–1330. 10.1016/j.sjbs.2020.11.056 33613062 PMC7878683

[45] Barbosa MC Jatobá A do Nascimento Vieira F : Cultivation of Juvenile Fat Snook ( *Centropomus parallelus* Poey, 1860) Fed Probiotic in Laboratory Conditions. *Braz. Arch. Biol. Technol.* 2011;54(4):795–801. 10.1590/S1516-89132011000400020

[46] Tocher DR : Metabolism and functions of lipids and fatty acids in teleost fish. *Rev.Fish. Sci.* 2003;11:107–184. 10.1080/713610925

[47] Tacon AGJ Matian M : Global overview on the use of fish meal and fish oil in industrially compounded aquafeeds: Trends and future prospects. *Aquaculture.* 2008, 28;1–4:146–158.

