# Supplementary material for: Fatty acid composition on diet and carcasses, growth, body indices and profile serum of Asian redtail catfish ( Hemibagrus nemurus) fed a diet containing different levels of EPA and DHA
Source: F1000Res. 2023 May 15;11:1409. Originally published 2022 Nov 30. [Version 2] doi: 10.12688/f1000research.126487.2 (PMC10233315; doi:10.12688/f1000research.126487.2)
Supplement: Peer review history

# Peer review: Reviewer response for version 2

**DOI:** 10.5256/f1000research.147971.r173674

**Published:** 2023-05-31

**Authors:** Christopher Marlowe Caipang

**Affiliations:** 1 Division of Biological Sciences, College of Arts and Sciences, University of the Philippines Visayas, Iloilo City, Philippines

The authors have addressed the comments/suggestions that were raised in the earlier version.

Is the work clearly and accurately presented and does it cite the current literature?

Yes

If applicable, is the statistical analysis and its interpretation appropriate?

Partly

Are all the source data underlying the results available to ensure full reproducibility?

Yes

Is the study design appropriate and is the work technically sound?

Yes

Are the conclusions drawn adequately supported by the results?

Yes

Are sufficient details of methods and analysis provided to allow replication by others?

Yes

Reviewer Expertise:

Aquaculture, fish health management, aquatic biotechnology

I confirm that I have read this submission and believe that I have an appropriate level of expertise to confirm that it is of an acceptable scientific standard.

# Peer review: Reviewer response for version 2

**DOI:** 10.5256/f1000research.147971.r173675

**Published:** 2023-05-24

**Authors:** Rudy Agung Nugroho

**Affiliations:** 1 Faculty of Mathematics and Natural Sciences, Mulawarman University, Samarinda, Indonesia

Present revised article is better than previous version. All suggestion and comment from reviewer have been applied in this version.

 This result of this study can be developed based on the molecular study in the next research.

 Based on my expertise, this article can be indexed.

Is the work clearly and accurately presented and does it cite the current literature?

Yes

If applicable, is the statistical analysis and its interpretation appropriate?

Partly

Are all the source data underlying the results available to ensure full reproducibility?

Yes

Is the study design appropriate and is the work technically sound?

Partly

Are the conclusions drawn adequately supported by the results?

Yes

Are sufficient details of methods and analysis provided to allow replication by others?

Partly

Reviewer Expertise:

Animal Physiology, Fish Nutrition

I confirm that I have read this submission and believe that I have an appropriate level of expertise to confirm that it is of an acceptable scientific standard.

# Peer review: Reviewer response for version 1

**DOI:** 10.5256/f1000research.138904.r157014

**Published:** 2023-01-31

**Authors:** Rudy Agung Nugroho

**Affiliations:** 1 Faculty of Mathematics and Natural Sciences, Mulawarman University, Samarinda, Indonesia

**Title**

- Good title.

**Abstract**

- Please add the results: Condition Factor (CF), Hepatosomatic Index (HSI), Viscerosomatic Index (VSI), and Liposomatic Index (LSI).

**Introduction**

- Brief background and aims.

**Materials and Methods**

- Table 1, at proximate composition NEE should be NFE (Nitrogen Free Extract).
- Table 1: Please describe the content of the vitamin and mineral mix.
- This species' health status was good. Please be specific on what categorize fish indicated as good in health status.
- Does the feeding rate of 3% was optimum for this fish? Or any previous experiment mentioned that 3% is optimum for this fish?
- It is stated in the methods that “Every 20 days, five fish samples were taken from each tarpaulin pond”. However, the results did not appear in the results section.

**Results and Discussion**

- The discussion of The link between digestive enzymes activity and the content of protein, carbohydrate, and fat contents which is related to the levels of GLU, TAG, HDL, and LDL in blood serum can be developed.

**Conclusion**

- Sufficient.

Current article is good and has a contribution in aquaculture field. However, the discussion need to be developed, based on the finding/results.

Is the work clearly and accurately presented and does it cite the current literature?

Yes

If applicable, is the statistical analysis and its interpretation appropriate?

Partly

Are all the source data underlying the results available to ensure full reproducibility?

Yes

Is the study design appropriate and is the work technically sound?

Partly

Are the conclusions drawn adequately supported by the results?

Yes

Are sufficient details of methods and analysis provided to allow replication by others?

Partly

Reviewer Expertise:

Animal Physiology, Fish Nutrition

I confirm that I have read this submission and believe that I have an appropriate level of expertise to confirm that it is of an acceptable scientific standard, however I have significant reservations, as outlined above.

# Response

**Published:** 2023-02-02

**Authors:** Netti Aryani

**Affiliations:** Universitas Riau, Indonesia

Abstract

1. We state that whole-body indices such as Condition Factor (CF), Hepatosomatic Index (HSI), Viscerosomatic Index (VSI), and Liposomatic Index (LSI) were not significant between diets experimental, so the result we do not write in quantity in the Abstract. On the other hand, the number of words in the abstract is limited to 300 words.

Methods

1. We agreed formula NEE should be changed to NFE (Nitrogen Free Extract) as written on the note under Table 1.
2. The vitamin mix contains vitamins A, 2,750 IU; Vit D, 550,000 IU; Vit E, 25,000 IU; Ca D-pantothenate, 25,000 mg, Mg, 25.000 mg; P, 50.000 mg.
3. A feeding rate of 3% has been optimal for the growth of *Hemibagrus nemurus* in this study, although some researchers reported a range of 3 - 8%.
4. Five fish samples were measured every 20 days, and their data were used for estimating the amount of feed given based on biomass weight (15 fish/pond) every 20 days. Hence, the results did not appear in the results section.

Result and Discussion

1. In this study, we did not analyze digestive enzyme activity, so the discussion related to the levels of GLU, TAG, HDL, and LDL in blood serum cannot get discussed yet; maybe in the future can be developed.

# Peer review: Reviewer response for version 1

**DOI:** 10.5256/f1000research.138904.r157015

**Published:** 2023-01-26

**Authors:** Christopher Marlowe Caipang

**Affiliations:** 1 Division of Biological Sciences, College of Arts and Sciences, University of the Philippines Visayas, Iloilo City, Philippines

This manuscript evaluated the effects of various levels of EPA and DHA on selected growth and body composition indices of Asian redtail catfish. Results showed that the incorporation of 6,300 mg of EPA+DHA/kg in the diets gave the best effects on whole-body carcass compositions, growth performance, and serum metabolites of the fish.

 Comments: This data obtained from this study is important in developing the aquaculture industry for this species of fish particularly in the establishment of commercial feeds. Comments and suggestions for the improvement of the manuscript are indicated below.

1. In the Conclusion (Abstract), why did the authors conclude that the level of 6,300 mg of EP+DHA in the feeds is the best for food safety? Please justify and support this claim with data from the work.
2. Please indicate the area or volume of the tarpaulin ponds so the stocking density could be determined.
3. Please justify the stocking of 15 fish per pond.
4. The use of commercial kits to measure blood parameters should be justified and authors should state whether these kits have been standardized for fish. 
5. Is there a need to write the formula of the different growth parameters in the text? Please explain. 
6. From Tables 2 - 5, the values of the SE were only placed in one column. Authors mentioned that they have triplicate in each treatment and 3 fish were sampled per replicate. Therefore, the number of fish per treatment should be 9. I suggest that each column (Control and D1-D4) should have its own SE. Kindly revise the tables accordingly.

I hope that the authors will take these suggestions/comments when they revise the manuscript.

Is the work clearly and accurately presented and does it cite the current literature?

Yes

If applicable, is the statistical analysis and its interpretation appropriate?

Partly

Are all the source data underlying the results available to ensure full reproducibility?

Yes

Is the study design appropriate and is the work technically sound?

Yes

Are the conclusions drawn adequately supported by the results?

Yes

Are sufficient details of methods and analysis provided to allow replication by others?

Yes

Reviewer Expertise:

Aquaculture, fish health management, aquatic biotechnology

I confirm that I have read this submission and believe that I have an appropriate level of expertise to confirm that it is of an acceptable scientific standard, however I have significant reservations, as outlined above.

# Response

**Published:** 2023-01-31

**Authors:** Netti Aryani

**Affiliations:** Universitas Riau, Indonesia

1. Although some studies recommended fat meat values between 0.5 and 1.0, no such guidelines exist for AI and TI. AI and TI values in the meat of Asian redtail catfish at 6,300 mg of EPA+DHA were 0.81 and 1.85. Foods with low AI and TI promote excellent human health, for example, by preventing human health coronary diseases. 
2. In the present study, the water volume of the tarpaulin ponds is 300 liters, with a stocking density of 15 fish per tarpaulin pond.
3. Primate Animal Study Center, IPB University-Bogor, has standardized commercial kits to measure blood fish parameters.
4. In the methods and calculations part, we revised the initial body weight formula, changed it to the final length (cm), and the final body weight (FBW) changed to the final weight (FW); we have presented in Table 4.
5. Standard Errors (SE) In Table 2-5, we wrote down the results of data analysis from multiple comparisons of the Post Hoc Tests for each analyzed parameter.
